# Experimental Study of the Moisture Resistance of Cement Mortar Using Pozzolan Materials and Calcium Stearate

**DOI:** 10.3390/ma17051014

**Published:** 2024-02-22

**Authors:** Jang Hyun Park, Chang Bok Yoon

**Affiliations:** 1 E-Moblilty Lab, Hankyong National University, Anseong 17579, Republic of Korea; parkjh@hknu.ac.kr; 2 Architectural Engineering, Seoil University, Seoul 02192, Republic of Korea

**Keywords:** calcium stearate, water repellent, nanosilica, diatomite

## Abstract

Nanosilica and diatomite are pozzolanic resources rich in SiO_2_. In this study, the purpose of this study was to improve the moisture resistance of the specimen by producing a mixed material using pozzolanic materials and calcium stearate and adding it to cement mortar while stirring. The results showed that the hydration reaction was not activated when calcium stearate adhered to the fine particles of nanosilica; it existed simply in the form of a filler inside the specimen. Diatomite, due to its atypical particles and porosity, may have greater water tightness than nanosilica because of the pozzolanic reaction in particles to which calcium stearate is not attached.

## 1. Introduction

Concrete has microscopic pores on the surface, which can cause damage such as water leaks when exposed to moisture. Damage due to the moisture infiltration of concrete occurs frequently, and the corrosion and expansion of reinforcing bars result in exposure to deterioration due to salt damage, freeze–thaw, etc., over time, and a decrease in durability.

Special processes are required to prevent moisture contact and penetration and improve the durability of concrete. In general, liquid-permeable paint or asphalt sheets are applied and attached to the concrete surface to install a separate waterproof protective layer to block moisture infiltration from the outside [1,2,3,4]. However, the installed protective layer may be destroyed due to external environmental factors such as cracks, impacts, and ultraviolet rays, creating a path for moisture to penetrate through the defect.

As a result, the damaged waterproofing layer may need to be partially repaired or completely rebuilt, causing inconvenience to users and requiring much time and cost [5]. Therefore, in order to compensate for these shortcomings, it is deemed necessary to conduct research on the development, and a performance evaluation, of moisture-resistant concrete. However, there is a lack of research on implementing moisture resistance in the concrete itself by using additive materials when mixing concrete instead of blocking moisture infiltration only on the concrete surface by applying materials to the surface. 

Therefore, in this study, in order to develop a cement mortar with a moisture-resistant performance, an admixture was prepared using pozzolanic materials such as nanosilica and diatomite and moisture-resistant calcium stearate, and experiments were performed by mixing them at a certain ratio when manufacturing cement mortar. Naseroleslami and Chari (2019) showed that cement mortar and paste mixed with calcium stearate enhanced durability performance such as capillary water absorption under non-static pressure conditions [6]. However, when calcium stearate is used, a mixture of at least 1% of the cement weight causes changes in its physical properties, such as a decrease in compressive strength. Calcium stearate is an inorganic mixture of saturated fatty acid metal salts that is already used to manufacture hydrophobic materials, and it has disadvantages, such as the need to be preprocessed before dispersion in a solid state and the difficulty of dispersion [7,8,9,10], but fatty acid metal salts are more insoluble and are expected to be better laminated on the surface of cement mortar (Choi et al., 2010) [11]. Thus, calcium stearate is expected to create hydrophobicity in cement mortar when applied to the surface or mixed internally. Nanosilica is an ultrafine particle of cement that causes a pozzolanic reaction during the hydration reaction. Thus, the compressive strength is expected to increase in the early stages. Previous studies using nanosilica have examined dispersion to improve compressive strength in terms of durability and demonstrated that nanosilica could improve strength through a hydration reaction in mixed cement [12,13,14,15,16]. Gaitero et al. and Ji [17,18] discovered that water penetration decreased when using nanosilica in mixing cement due to the creation of a watertight structure with the filling of hydration products in the transition zone resulting from the pozzolanic reaction.

Diatomite is a very light and porous pozzolanic material consisting of deposited phytoplankton shells with unique physical properties, such as high porosity, water permeability, surface area, chemical stability, water absorption, and thermal resistance, owing to the irregular shapes and porous structure of diatom particles and their fragments. The principal component of the shell is opal (SiO_2_·nH_2_O), which is amorphous silica. The content of SiO_2_, the principal component, is approximately 70–80%, with the remainder consisting of Al_2_O_3_, Fe_2_O_3_, CaO, and MgO. The particle size is approximately 1 mm and takes various forms depending on the species [19]. Recently, diatomite has been used as a concrete admixture after firing to improve the durability and strength efficiency of concrete [20,21,22,23,24]. 

**Figure 1:**
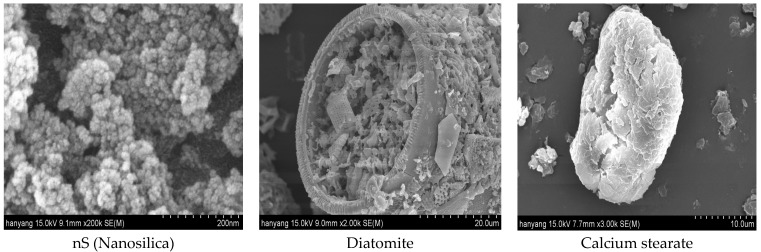
Sem analysis.

Calcium stearate is a powder-type material and has its own hydrophobicity, and nanosilica and diatomite have a high content of SiO_2_ and also have amorphous pores. Therefore, a pozzolanic reaction occurs during the hydration stage, which has the advantage of increasing water tightness and improving durability [25,26]. Using the pores of nanosilica and diatomite, calcium stearate, a powder-like material that can reduce moisture resistance, was attached to the inside of the pores to produce a moisture-resistant mixed material, and its physical performance and durability were evaluated. Figure 1 shows SEM images of nanosilica, diatomite, and calcium stearate used in the experiment.

## 2. Materials and Methods of the Experiment

### 2.1. Materials

The cement used in this experiment is a domestic type 1 ordinary Portland cement specified in KS F 5201 [27] and ASTM C 150 [28], with a density of 3.15 g/cm^3^ and 3000 cm^2^/g. The nanosilica used is available in the domestic market (S teck company, Ansan, Gyeonggi, Republic of Korea), as well as the diatomite (Daejung company, Seoul, Republic of Korea). For the fine aggregate, a product that complies with ISO 679:2009 [29] was used, and the washed aggregate with a fineness modulus of 2.8 was used. The calcium stearate used in this experiment was obtained from the reaction of stearic acid and lime and exhibited lubricating and water-repellent properties. More specifically, it is a white powder, available in the domestic market (Daejung company, Seoul, Republic of Korea), that has a lower density than cement and a pH close to slightly alkaline or neutral. The chemical compositions of the cement, nanosilica, diatomite, and calcium stearate are shown in Table 1 and Table 2 below.

### 2.2. Overview and Methods of the Experiment

#### 2.2.1. Preparation of Hydrophobic Powder

This experiment aimed to produce moisture resistance inside cement mortar using nanosilica and diatomite, which have an excellent pozzolanic reactivity, and using calcium stearate to enhance insolubility. Owing to its unique insolubility, calcium stearate does not easily dissolve in contact with cement mortar mixing water. Accordingly, it is expected to result in bleeding. Thus, an impregnation method was applied to prevent the detachment of calcium stearate by attaching calcium stearate to the surface of each pozzolan powder using nanosilica and diatomite as carriers. To this end, calcium stearate was dissolved using isopropylene as a solvent, after which nanosilica and diatomite were added and mixed evenly in an ultrasonic stirrer for approximately 3 h so that the nanosilica, diatomite, and calcium stearate could adhere. The mixture was then completely dried for 3 days in natural conditions and 2 days at 70 °C. Subsequently, it was powdered. The powders that passed the standard screen size of 150 μm were used to obtain an even fineness. Finally, the completed powders were named NSC and DTC. The experimental formulation table is shown in Table 3.

#### 2.2.2. Method of Specimen Production and Experiment

Mixing was performed according to KS L 5109 [31] for a physical performance evaluation and microstructure analysis. For mixing, cement and binding powder were dry-mixed by adding 1% and 3% of the cement weight ratio, respectively. Next, mixing water was added and stirred for 30 s at a single speed, and then sand was added and mixed for 30 s at two speeds. It was stirred slowly for 30s at a speed of 140 ± 5 r/min, then left for 90 s, and finally stirred for 60 s at two speeds of 285 ± 10 r/min. For the compressive strength and microstructure analysis, water absorption test, and chloride penetration resistance test, the specimen was cast into a square mold 50 mm on the side and a round cylinder of ⏀100 × 150 mm, which was demolded after 1 day and cured in water. The experiments were then conducted at the ages of 7 and 28 days. The mix proportions for this experiment are shown in Table 3.

Furthermore, this study measured the activity factor using the compressive strength at the age of 28 days and the compressive strength according to KS L 5105 [32] and KS L 5405 [33] to determine the physical properties of the specimen. Afterward, a thermal analysis (TGA) was conducted, and a scanning electron microscope (SEM) was used for microanalysis, and water absorption and permeability resistance tests were performed at the ages of 7 and 28 days based on KS F 4919 [34] to evaluate the internal moisture resistance of the specimen. In addition, the chloride ion penetration resistance was tested according to ASTM C 1202 [35] “Standard Test Method for Electrical Indication of Concrete’s Ability to Resist Chloride Ion Penetration” (ASTM 1993) and KS F 2711 [36] “Standard test method for resistance of concrete to chloride ion penetration by electrical conductance” (KSA 2017).

## 3. Experimental Results

### 3.1. Thermal Analysis Measurement

**Figure 2:**
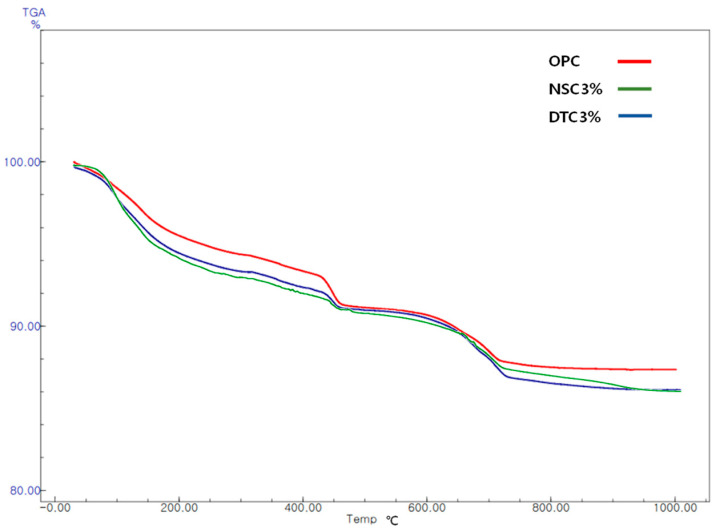
Results of thermogravimetry differential thermal analysis.

Ca(OH)_2_ is thermally decomposed at approximately 450–550 °C. A thermal analysis of the reference mortar and NSC3% and DTC3% was conducted after 7 days to determine the weight loss of the specimen using mass reduction and quantitatively evaluate the amount of Ca(OH)_2_ produced in the hydrate. The TGA measurement results are shown in Figure 2.

The amount of Ca(OH)_2_ produced from the reference mortar was 13.4%, whereas the amounts from DTC3 and NSC3% were 2.51% and 12.05%, respectively. In the NSC specimen, the lowest mass reduction rate was determined through the endothermic peak in the section where Ca(OH)_2_ was decomposed. Nanosilica promotes the hydration of cement at the early stage of the hydration reaction, consumes calcium hydroxide (CH) due to its high pozzolanic reaction, and then converts it to calcium silicate hydrate (CSH) gel to improve the mechanical properties of concrete. However, the pozzolanic reaction was reduced when insufficient CH was produced, as under the conditions of this experiment. The NSC specimen using nanosilica does not adhere to the surface when combined with calcium stearate, whereas calcium stearate adheres in the form of a coating due to the shape of the fine particles of nanosilica, without causing a pozzolanic reaction. Moreover, in the specimen using diatomite, the CH generated by the hydration reaction of cement and the SiO_2_ present in large amounts in diatomite seems to have partially compensated for the loss of compressive strength following the mixing of calcium stearate due to the pozzolanic reaction.

### 3.2. Compressive Strength and Activity Factor

**Figure 3:**
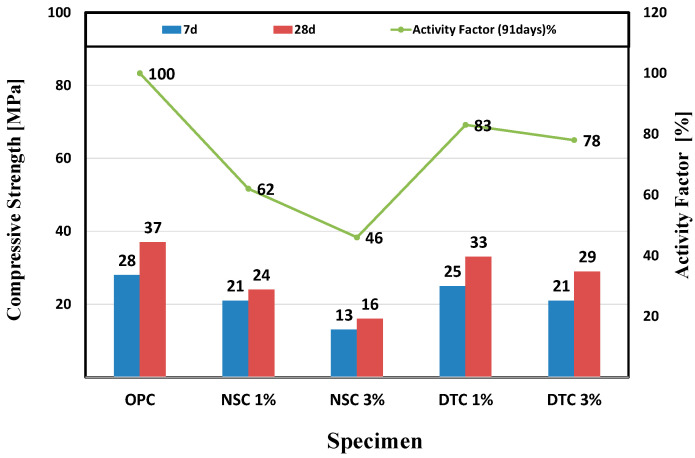
Results for compressive strength and activity factor.

The following values were determined when measuring the 7-day and 28-day compressive strength. OPC, the control group, showed 28 MPa on the 7th day and 37 MPa on the 28th day, whereas NSC, which combined nanosilica and calcium stearate, exhibited a significantly lower compressive strength. The 7-day and 28-day compressive strengths of the specimen mixed with a weight ratio of 1% were 21 and 24 MPa, respectively, and those of the specimen mixed with a weight ratio of 3% were 13 and 16 MPa, respectively. The compressive strength, activity factors, and the equation used to evaluate the activity factor used in Equation (1) are shown in Figure 3 below.

(1) $$As=\frac{C_{1}}{C_{2}}\;\times \;100(\%)$$

where

As: activity factor (%);
C_1_: average compressive strength of the specimen replacing admixture (MPa);
C_2_: average compressive strength of OPC specimen (MPa).

The compressive strength increased when nanosilica was mixed because the size of the basic particles of nanosilica was 10–20 nm, and the fine particles reduced the porosity of concrete due to the void filling and particle distribution and increased the pozzolanic reaction with Ca(OH)_2_, thereby generating CSH [37,38]. However, in this experimental condition, the dissolved calcium stearate surrounded the entire particle when attached to nanosilica; thus, the high content of SiO_2_ in nanosilica could not cause a pozzolanic reaction and delayed the hydration reaction when only calcium stearate was mixed, reducing the increase in compressive strength because the transition area representing the interface between cement paste and particles of aggregate was expanded. This consequently hindered a uniform dispersion force [39,40]. The DTC specimens that combined diatomite and calcium stearate showed an increase in activity of up to 83% compared to the reference mortar. In contrast to the NSC case, calcium stearate was attached within the pores due to the characteristics of the internal pore structure of diatomite. Thus, some of the diatomite compensated for the compressive strength that was reduced through the pozzolanic reaction.

### 3.3. Water Absorption Measurement

**Figure 4:**
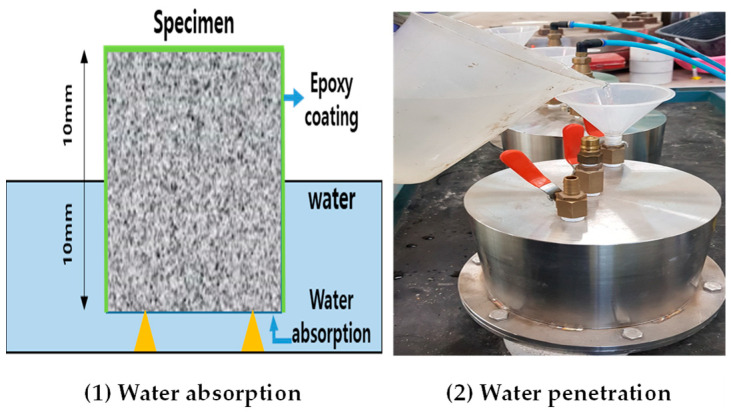
Water resistance test.

Water absorption was measured at 7 days and 28 days based on KS F 4919 [34]. Absorption is typically measured by immersing the lower surface of a 70 mm × 70 mm × 20 mm test specimen coated with a waterproofing agent in water; however, in this experiment, the central part of a 50 mm × 50 mm × 50 mm square mortar plate was cut, and the cut surface was immersed to measure water absorption inside the mortar plate specimen. The initial mass (W_0_) was measured after applying epoxy to the remaining surfaces, except the lower bottom, where the cut surface was immersed in water. The immersed surface was taken out after 24 h, and the mass (W_1_) was measured after gently wiping the surface. Absorption was determined by calculating the means of the three specimens according to Equation (2). Moreover, a ⏀100 × 200 circular test piece was divided into four lengthwise sections to conduct a water penetration test in pressurization. Subsequently, a water permeability test was conducted using a specimen with a height of 50 mm on the inner part. The experiment was conducted under the atmospheric pressure of 0.3 N/mm^2^ to allow moisture to permeate the specimen, whereas the weight was measured after 3 h of pressurization. The equation used to measure moisture absorption is shown in Equation (2), and the hydrostatic pressure and atmospheric pressure are shown in Figure 4.

Absorption(g) = W_1_ − W_0_ (2)

where

W_0_: mass before measurement (g);
W_1_: mass after measurement (g).

The water absorption measurements revealed that the reference mortar showed a water absorption of 2.7 g, whereas the NSC specimen using nanosilica showed slightly less water absorption than the DTC specimen using diatomite. However, when a pressure of 0.3 N/mm^2^ was applied, the NSC specimen showed an increase in absorption as the pressure increased compared to the reference mortar. Specifically, the NSC specimen only showed moisture resistance due to the characteristics of the calcium stearate surrounding the surface of nanosilica. The DTC specimen showed a lower compressive strength than the reference mortar; however, in contrast to the NSC specimen, the characteristics of calcium stearate attached inside the pores of the diatomite and the hydration reaction due to the pozzolanic reaction had a simultaneous effect that enhanced the moisture resistance performance. Except for the reference mortar, the pore structure was not improved by the filling of the capillary pores with the hydration product formed by the pozzolanic reaction. More precisely, moisture resistance was found to be due to the characteristics of calcium stearate. The results are presented in Table 4 and Table 5, respectively.

The water absorption measurements revealed that the reference mortar showed a water absorption of 2.7 g, whereas the NSC specimen using nanosilica showed slightly less water absorption than the DTC specimen using diatomite. However, when a pressure of 0.3 N/mm^2^ was applied, the NSC specimen’s water absorption increased as the pressure increased compared to the reference mortar. Specifically, the NSC specimen only showed moisture resistance due to the characteristics of the calcium stearate surrounding the surface of nanosilica. The DTC specimen showed a lower compressive strength than the reference mortar; however, in contrast to the NSC specimen, the characteristics of the calcium stearate attached inside the pores of the diatomite and the hydration reaction due to the pozzolanic reaction had a simultaneous effect that enhanced the moisture resistance performance. Except for the reference mortar, the pore structure was not improved by the filling of the capillary pores with the hydration product formed by the pozzolanic reaction. More precisely, moisture resistance was found to be due to the characteristics of calcium stearate.

### 3.4. Measurement of Chloride Ion Penetration Resistance

**Figure 5:**
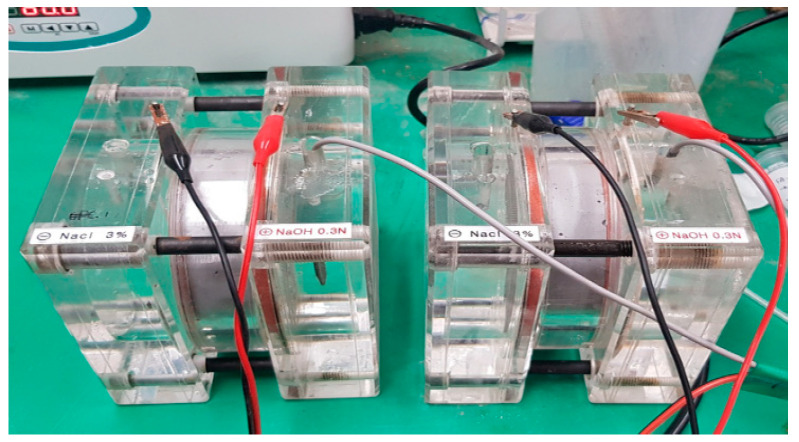
Testing of RCPT [41].

To measure the chloride ion penetration resistance of each specimen as chloride ions moved, along with water penetration, an experiment was conducted according to ASTM C 1202 (ASTM 1993) and KS F 2711 [34,35]. The experimental measurement is shown at Figure 5.

**Figure 6:**
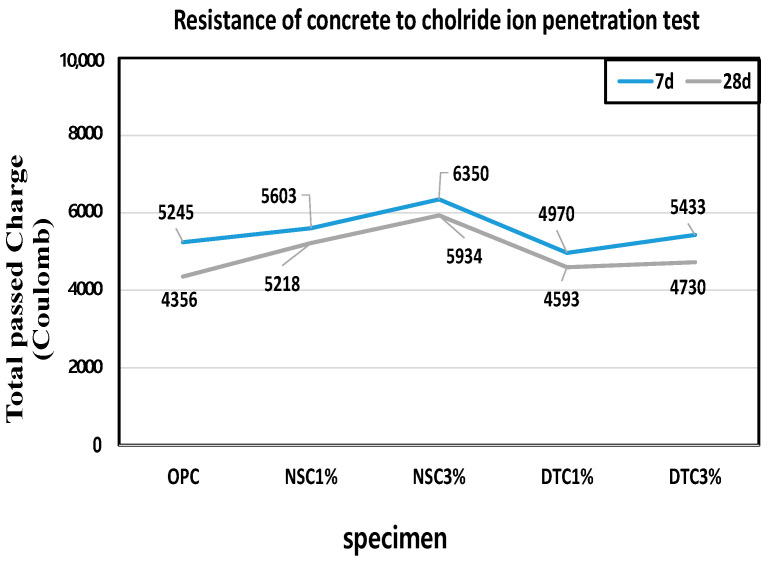
Mesurement of RCPT.

The specimens were retained in a desiccator under vacuum for 3 h. Subsequently, they were immersed in distilled water for 18 ± 2 h until the interiors of the specimens were completely saturated. The cathode of the applied voltage cell was then filled with a 3.0% NaCl solution and the anode with a 0.3 N NaOH solution. A direct current voltage of 60 V was maintained on both sides of the applied voltage cell and the current was recorded every 30 min for 6 h. Equation (3) and Table 6 were used to calculate the total charge that passed and the results of coulombs (C) are shown at Figure 6.

(3) $$Q\;=\;900(I_{0}+2I_{30}+2I_{60}+\ldots 2I_{300}+2I_{330}+I_{360})$$

where

Q: total passing charge;
I_0_: current immediately after starting test with applied voltage;
I_360_: current 360 min after applying voltage.

**Table 6:** Evaluation according to chloride ion penetration [41].

Coulombs (C)	Permeability
>4000	High
2000–4000	Normal
1000–2000	Low
100–1000	Very low
<100	Negligible

In the 28-day measurement, the OPC, the reference mortar in this experiment, showed a high value of approximately 4300 coulombs, whereas the C values of NSC 1% and 3% specimens were 5600 and 6300, respectively, somewhat higher than DTC 1% and 3% at 4900 and 5400. Calcium stearate generally exhibits water penetration resistance because a hydrophobic wax compound is created through contact with moisture. However, the specimen lacks water tightness, as the calcium stearate coating film formed on the nanosilica particles inhibits the formation of hydrates in the cement mortar, causing water penetration. The reaction of calcium stearate and cement may decrease chloride penetration resistance due to the deterioration in compressive strength [42]. The results for the samples with calcium stearate revealed no improvement in the pore structure inside the cement mortar.

### 3.5. Scanning Electron Microscope (SEM)

**Figure 7:**
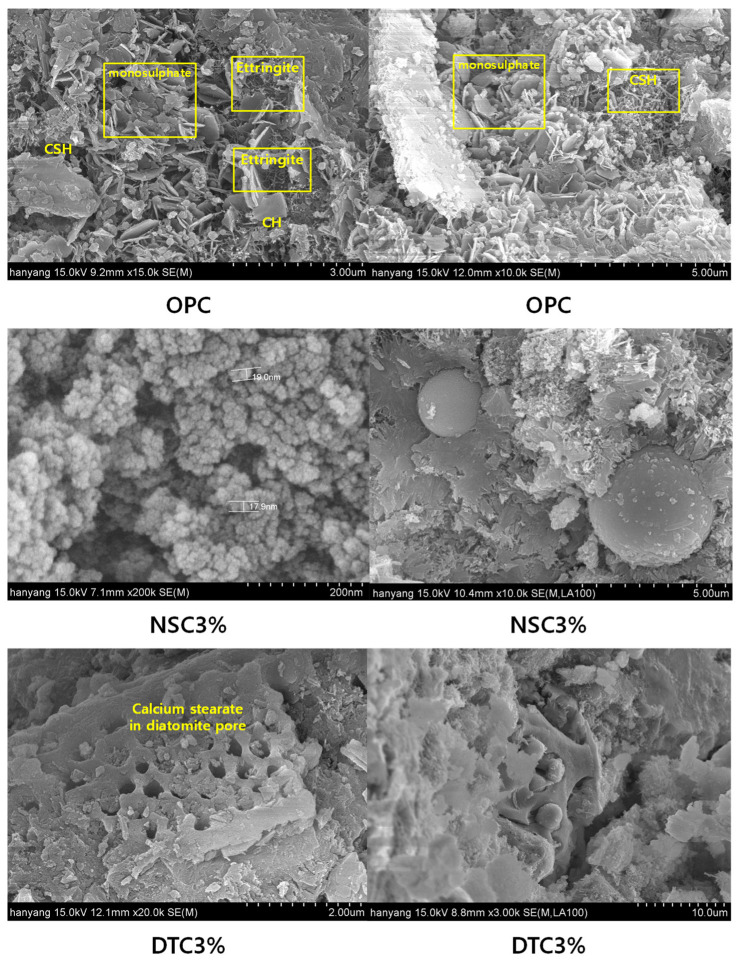
Result of SEM analysis.

In the OPC specimen, hydration products such as CSH and CH, Ca(OH)_2_ were evenly distributed according to the hydration process, whereas, in the NSC specimen, the hydration reaction was observed only in some parts. In the SEM image, the size of the nanosilica particles was approximately 17–18 nm, which was ultrafine. Thus, the dissolved calcium stearate seemed to have surrounded the aggregated particles instead of being attached to the nanosilica particles. Accordingly, the limited bonding between calcium oxide and nanosilica, with its high SiO_2_ content, seems to have inhibited the pozzolanic reaction. As in OPC, hydration products were found in the DTC specimen. Calcium stearate attached inside the pore structure of diatomite, resisting water that had penetrated. However, its compressive strength was less than that of OPC, the reference mortar, due to the uneven adhesion of calcium stearate to diatomite and the delay in the production of hydration because even dispersion was not secured within the cement mortar. The sem measurement image was shown at Figure 7.

## 4. Conclusions

This study used nanosilica and diatomite, which can serve as pozzolanic admixture materials, to endow concrete with a hydrophobic property through adhesion with calcium stearate, selected for its moisture resistance. It was added when mixing cement mortar, and, subsequently, we tested the internal moisture resistance. The following results were obtained:

(1) The results of the literature review showed that mixing in only nanosilica can increase compressive strength. However, the fine particles of nanosilica are coated with liquefied calcium stearate, thereby inhibiting the pozzolanic reaction under the conditions of this experiment.
(2) The moisture resistance measurement results were the best for the NCS3% specimen, which was rich in calcium stearate. It exhibited the best results under hydrostatic pressure, whereas its permeability was the highest under pressurization. As a simple filler, it can resist some moisture; however, water tightness cannot be expected due to the chemical reaction of nanosilica.
(3) The chloride ion penetration resistance measurements also demonstrated that the increase in the amount of mixing admixture to which calcium stearate was attached led to an increase in the total charge that passed.
(4) Diatomite, with its atypical particles and porosity, seems to have secured water tightness better than nanosilica because of the pozzolanic reaction in particles to which calcium stearate was not attached.

It is expected that it will be possible to determine the feasibility of using porous pozzolanic admixtures such as diatomite as a carrier for developing admixtures with moisture resistance. Further research should combine hydrophobic substances using pozzolanic powder with porosity and compensate for the reduction in compressive strength.

## Figures and Tables

**Table 1:** Chemical composition of materials [30].

Name	Chemical Composition (%)							
	SiO_2_	Al_2_O_3_	Fe_2_O_3_	CaO	MgO	SO_3_	K_2_O	L.O.I
OPC	19.29	5.16	2.87	61.68	4.17	2.53	0.89	
Diatomite	91.56	1.34	3.83	1.0	0.2	0.01	0.06	
NanoSilica	99.46	0.03	0.02	0.07	0.01	0.24	0.01	0.17

**Table 2:** Chemical composition of metallic salts.

Name	Chemical Composition of Metallic Salts				
	Chemical Formula	Density (g/cm^3^)	pH	Melting Point	Molecular Weight (g/mol)
Calcium stearate	C_36_H_70_CaO_4_	1.10	7−9	147−149	607

**Table 3:** Experimental mixing table of cement mortar.

Specimen	W/B	C	S	W	NSC	DTC
OPC	50%	510	1530	255	-	
NSC 1%				257.5	5.1	
NSC 3%				260.1	10.2	
DTC 1%				257.5		5.1
DTC 3%				260.1		10.2

**Table 4:** Results of water absorption test.

	Specimen [28 Days]				
	OPC	NSC 1%	NSC 3%	DTC 1%	DTC 3%
0 H (g, W_0_)	133.4	137.7	131.5	134.9	139.5
24 H (g, W_1_)	136.1	140.1	133.1	137.6	141.8
Absorption (g)	2.7	2.4	1.6	2.7	2.3

**Table 5:** Results of water penetration test.

	Specimen [28 Days]				
	OPC	NSC 1%	NSC 3%	DTC 1%	DTC 3%
Weight (before testing)	855.8	869.5	850.7	862.8	860.4
Weight (after the test)	870.9	888.4	875.3	879.1	876.1
Absorption (g)	15.1	18.9	24.6	16.3	15.7

## Data Availability

Data are contained within the article.
